# Insights Into the Molecular Mechanisms of Late Flowering in *Prunus sibirica* by Whole-Genome and Transcriptome Analyses

**DOI:** 10.3389/fpls.2021.802827

**Published:** 2022-01-25

**Authors:** Wanyu Xu, Wenquan Bao, Huimin Liu, Chen Chen, Haikun Bai, Mengzhen Huang, Gaopu Zhu, Han Zhao, Ningning Gou, Yixiao Chen, Lin Wang, Ta-na Wuyun

**Affiliations:** ^1^State Key Laboratory of Tree Genetics and Breeding, Non-timber Forest Research and Development Center, Chinese Academy of Forestry, Zhengzhou, China; ^2^Kernel-Apricot Engineering and Technology Research Center of State Forestry and Grassland Administration, Zhengzhou, China; ^3^Key Laboratory of Non-timber Forest Germplasm Enhancement and Utilization of National Forestry and Grassland Administration, Zhengzhou, China; ^4^College of Forestry, Inner Mongolia Agricultural University, Hohhot, China

**Keywords:** TPP, Tre6P, TPS, floral transition, GWAS, ceRNA

## Abstract

Freezing during the flowering of *Prunus sibirica* is detrimental to fruit production. The late flowering (LF) type, which is delayed by 7–15 days compared with the normal flowering (NF) type, avoids damages at low temperature, but the molecular mechanism of LF remains unclear. Therefore, this study was conducted to comprehensively characterize floral bud differentiation. A histological analysis showed that initial floral bud differentiation was delayed in the LF type compared to the NF type. Genome-wide associated studies (GWAS) showed that a candidate gene (PaF106G0600023738.01) was significantly associated with LF type. It was identified as *trehalose-6-phosphate phosphatase* (*PsTPPF*), which is involved in trehalose-6-phosphate (Tre6P) signaling pathway and acts on floral transition. A whole-transcriptome RNA sequencing analysis was conducted, and a total of 6,110 differential expression (DE) mRNAs, 1,351 DE lncRNAs, and 148 DE miRNAs were identified. In addition, 24 DE mRNAs related with floral transition were predicted, and these involved the following: three interactions between DE lncRNAs and DE mRNAs of photoperiod pathway with two mRNAs (*COP1*, PaF106G0400018289.01 and *CO3*, MXLOC_025744) and three lncRNAs (*CCLR*, LTCONS_00031803, *COCLR1*, LTCONS_00046726, and *COCLR2*, LTCONS_00046731); one interaction between DE miRNAs and DE mRNAs with one mRNA, encoding *trehalose-6-phosphate synthase* (*PsTPS1*, PaF106G0100001132.01), and one miRNA (miRNA167h). Combined with the expression profiles and Tre6P levels, functions of *PsTPPF* and *PsTPS1* in Tre6P regulation were considered to be associated with flowering time. A new network of ceRNAs correlated with LF was constructed, and it consisted of one mRNA (*PsTPS1*), one lncRNA (*TCLR*, LTCONS_00034157), and one miRNA (miR167h). This study provided insight into the molecular regulatory mechanism of LF in *Prunus sibirica*.

## Introduction

*Prunus sibirica* is a stone fruit and woody oil plant that has excellent resistance to extreme environment conditions and is both economically and ecologically valuable (Zhang et al., 2006; Wang et al., 2017). It is an early flowering phenotype species, and its yield is susceptible to low-temperature weather during flowering. When *P. sibirica* flowers late, damage is avoided, and apricot yield is ensured; however, only few studies have investigated the associated mechanism (Wang et al., 2019).

Floral transition time is an important factor affecting the flowering of plants, and it further affects their life cycle. It is controlled by a complex network of numerous genes involved in genetic pathways, and several floral transition genes have been reported, including *CONSTAN* (*CO*) in photoperiod pathway (Suarez-Lopez et al., 2001), DELLA proteins in gibberellin (GA) pathway (Hou et al., 2008), *VRNs* in vernalization pathway (Levy et al., 2002), and *SQUAMOSA promoter binding protein-like* (*SPLs*) in age pathway (Wu and Poethig, 2006), and *trehalose-6-phosphate synthase* (*TPS1*) in trehalose-6-phosphate (Tre6P) signaling pathway (Wahl et al., 2013).

Using the sequencing technique process, genome-wide associate studies (GWASs) have been conducted to identify candidate genes associated with important traits in perennial fruit trees. For example, studies have investigated *Prunus persica* and *Pyrus pyrifolia* to identify fruit quality and phonological traits (Cao et al., 2016; Zhang et al., 2021), *Prunus armeniaca* to analyze pathogen resistance (Mariette et al., 2016), *Prunus mume* to determine floral traits (Zhang et al., 2018), and *Malus domestica* for fruit quality traits (Duan et al., 2017). Recently, several candidate genes associated with flowering time in *P. persica* (Li et al., 2019), *Helianthus annuus* (Bonnafous et al., 2018), and *Brassica napus* (Huang et al., 2021) have been identified by GWASs.

Whole transcriptome RNA sequencing analyses have been performed to investigate non-coding RNAs (ncRNAs), which participate in multiple biological and abiotic functions. NcRNAs are divided into two categories by length, small RNAs and long non-coding RNAs (lncRNAs). Notably, ncRNAs played important roles in the control of flowering; for example, two well-known lncRNAs, *COLD-INDUCED LONG ANTISENSE INTRAGENIC RNA* (*COOLAIR*) and *COLD-ASSISTED INTRONIC NON-CODING RNA* (*COLDAIR*) acted on vernalization process associated with controlling flowering time by silencing the transcription of *FLC* (Liu et al., 2010; Heo and Sung, 2011). The studies of Wu and Poethig (2006) and Wu et al. (2009) first reported that miR156 and miR172 controlled floral transition in *Arabidopsis thaliana*, with opposite regulatory effects. Several micro RNAs (miRNAs) involved in floral transition have also been studied, such as miR159 (Li et al., 2013), miR168 (Xian et al., 2014), milR169d (Xu et al., 2014), and miR390 (Fahlgren et al., 2006; Garcia, 2008). Competing endogenous RNAs (ceRNAs) have been found to be an RNA molecule class that possesses at least one common miRNA response element (MRE) that is accessible to miRNA binding (Salmena et al., 2011). Furthermore, protein-coding RNAs and ncRNAs, such as pseudogene transcripts, lncRNAs, and circular RNAs (circRNAs), have been found to communicate and co-regulate with each other by competitively combining same miRNAs; these are considered to be ceRNAs, and they possibly participate in biology processes (Tay et al., 2014). ceRNAs have been investigated in studies focusing on human disease in relation to their functions of competitive expression with a disease-causing gene (Lü et al., 2016; Liang et al., 2018). However, only a few studies have focused on the ceRNA network and association between ceRNA networks and flower traits in plants; for example, studies have investigated ceRNA networks in *Solanum lycopersicum* (Yang et al., 2019) and *Brassica campestris* (Liang et al., 2019).

To elucidate the molecular mechanism of late flowering (LF) in *P. sibirica*, we conducted a GWAS to identify the loci and candidate genes associated with LF. We also conducted an integrated analysis of mRNAs, lncRNAs, and miRNAs to construct ceRNAs networks by whole transcriptome RNA-sequencing. This study provides new insight into the genetic regulation of LF mechanisms in *P. sibirica*.

## Materials and Methods

### Plant Materials

A total of 66 *P. sibirica* accessions were used in this study, which grown in the Inner Mongol Forest Seed Breeding Center in Horinger County within the Inner Mongolia Autonomous Region. Full bloom date (FBD) was defined as when up to 50% of the flowers opened (Pérez-Pastor et al., 2004), and the FBD of all the accessions were measured from 2015 to 2017 (Supplementary Table 1). Fresh young leaves of *P. sibirica* accessions were collected, immediately frozen in liquid nitrogen, and stored in a refrigerator at −80°C prior to conducting the GWAS. A minimum of 5 g (fresh weight) of floral buds collected from LF and normal flowering (NF) accessions were respectively used to conduct histological analyses and whole-transcriptome RNA sequencing.

### Sampling and Observations of Tissue Morphology in Paraffin Sections

Floral buds (a minimum of 20 floral buds from each sample) from the LF and NF types were sampled every 10 days from June 20 to July 10, 2017, every 3 days from July 10 to October 11, and every 7 days from October 11, 2017 to April 3, 2018. After fixation in an FAA fixative solution for 24 h, the buds were kept in a 70% alcohol solution under 4°C. The materials were then subjected to alcohol dehydration, xylene treatment, waxed treatment, embedding, and slicing at a thickness of 6–10 μm. Samples were subsequently dyed with Fast Green and counterstained with safranin. Tissues were observed, and images were obtained under an optical microscope, Olympus BX-51 (Olympus Optical, Tokyo, Japan).

### DNA Preparation, Sequencing, Sequence Alignment, and SNP Calling

A total of 66 *P. sibirica* accessions were used in this study, of which 41 have been used in our previous study (Genome Sequence Archive, PRJCA001987) and 25 were newly sequenced. The leaf DNA of 25 *P. sibirica* accessions was extracted with CTAB methods, and sequence libraries were constructed with fragment sizes of up to 300 bp. The libraries were then sequenced on an Illumina HiSeq X Ten platform. After sequencing, clean reads were obtained using fastp (version 1.12.6, default parameters) by removing low-quality reads with adapters at both or either end, and with the number of N bases accounting for more than 5% (Chen et al., 2018b). The genome sequence data of 25 *P. sibirica* accessions were submitted to Genome Sequence Archive (PRJCA006925).

Whole-genome sequences for each accession were mapped to the *P. sibirica* “F106” reference genome (Genome Database for Rosaceae, tfGDR1049) using BWA-MEM (version 0.7.17, -K 100000000 -v 3 -Y) (Li, 2013). Duplicates were then marked using the genome analysis toolkit (GATK) MarkDuplicates (version 4.1.2.0, –VALIDATION_STRINGENCY SILENT –OPTICAL_DUPLICATE_PIXEL_DISTANCE 2500 –ASSUME_SORT_ORDER “queryname”) (McKenna et al., 2010). A GATK Haplotype Caller was used to detected SNP variant calls (version 4.1.2.0, -ERC GVCF). The GATK (version 4.1.2.0) was used for hard filtering with the following parameters: -filter “QD < 2.0” –filter-name “QD2” -filter “QUAL < 30.0” –filter-name “QUAL30” -filter “SOR > 3.0” –filter-name “SOR3” -filter “FS > 60.0” –filter-name “FS60” -filter “MQ < 40.0” –filter-name “MQ40”-filter “MQRankSum < -12.5” –filter-name “MQRankSum-12.5” -filter “ReadPosRankSum < -8.0” –filter-name “ReadPosRankSum-8.” All the SNPs were annotated for potential coding effects using ANNOVAR (version 2018-04-16, default parameters) (Yang and Wang, 2015).

### Genome-Wide Association Study

To improve the statistical power of the analysis, a set of bi-allelic SNPs with missing rates of less than0.2 and minor allele frequency (MAF) of >0.05 were obtained to conduct subsequent analyses. Principal component analysis was performed using Smartpca (Zhang, 2009). A distance matrix was generated with VCF2D (version 1.0), and a neighbor-joining tree was constructed with 1,000 bootstraps using TreeBeST (version 1.9.2). A mixed linear model (MLM) program in Efficient Mixed-Model Association Expedited (EMMAX, version beta-07Mar2010, default parameters) (Kang et al., 2010) software was used for the GWAS. The results were visualized as Manhattan plots and Q–Q plots using the R package “qqman” (Turner, 2014). We defined the cutoff of associated signals as the Bonferroni test threshold (Gao et al., 2010), which was set as0.05/total SNPs [−log10 (0.05/2598398) = 7.71]. Candidate regions were defined as ±10 kilobase (kb) on either side of significant association peaks. Exons and introns of the candidate genes were drawn with Illustrator for Biological Sequences (IBS, version 1.0.3) (Liu et al., 2015) using genomic DNA sequences.

### Total RNA Extraction, lncRNA and small RNA Library Construction, and Sequencing

The total RNA was extracted from floral buds of the LF and NF types obtained on July 10, 2017 using a ethanol precipitation protocol and a CTAB-PBIOZOL reagent. The results were qualified and then quantified using a NanoDrop and an Agilent 2100 (Thermo Fisher Scientific, Waltham, MA, United States) bioanalyzer. Two biological replicates were analyzed for each RNA sample.

For the long non-coding RNA library, total RNA was treated with Ribo-Zero™ Magnetic Kit (plant leaf) (epicenter) to deplete rRNA. The transcription was reversed, and the adapters were ligated. The qualified libraries were sequenced pair end on the Hiseq X-Ten (BGI-Shenzhen, Shenzhen, China) platform. After sequencing, clean reads were obtained by removing low-quality reads and reads, with the number of N bases accounting for more than 10%. The clean reads generated by high-throughput sequencing were mapped on the *P. sibirica* genome using the HISAT2 software (version 2.0.4) (Kim et al., 2015), and the reads mapped on the genome were assembled into transcripts using the StringTie software (version 1.0.4) (Pertea et al., 2015). The raw data of mRNA sequencing were submitted to the Genome Sequence Archive (PRJCA001987).

Small RNA library was prepared with 1 μg total RNA for each sample. Total RNA was purified by electrophoretic separation on a 15% urea denaturing polyacrylamide gel electrophoresis (PAGE) gel, and small RNA regions corresponding to the 18–30 nt bands in the marker lane (14–30 ssRNA Ladder Marker, TAKARA, Dalian, China) were excised and recovered. The adaptors were then ligated, transcribed into cDNA, and purified. Final ligation PCR products were sequenced using the BGISEQ-500 platform (BGI-Shenzhen, Shenzhen, China). The small RNA sequencing raw data were submitted to the Genome Sequence Archive (PRJCA006925).

### Functional Annotation and Expression Calculation

The CPC (Kong et al., 2007), txCdsPredict, and CNCI (Sun et al., 2013) software, and Pfam database (Finn et al., 2016) were used to predict the coding ability of the transcript and distinguish between mRNA and lncRNA. The transcripts were then aligned to the reference sequence with Bowtie2 (Langmead and Salzberg, 2012), and expression levels were calculated using fragments per kilobase per million (FPKM) with RSEM (Li and Dewey, 2011). The transcripts were subsequently aligned against the Kyoto Encyclopedia of Genes (KEGG) database (Kanehisa et al., 2007) and Gene ontology (GO) database (Ye et al., 2006). miRA (Evers et al., 2015) was used to predict novel miRNAs by exploring the characteristic hairpin structure of miRNA precursor. The small RNA expression levels were calculated by transcripts per kilobase million (TPM; ’t Hoen et al., 2008).

Significant differentially expressed genes, including mRNAs and lncRNAs, were selected with | Log2Ratio| ≥ 1.00 and adjusted *p*-value ≤ 0.001 by DEG-seq (Storey, 2003; Wang et al., 2010). The *P* values were corrected using the Benjamini–Hochberg method. DESeq2 (Love et al., 2014) with the default threshold “| Log2Ratio| ≥ 1.00 and adjusted *p*-value ≤ 0.1” was used to detected significant differentially expressed miRNAs. Heatmaps were visualized with TBtools (version 1.098661) (Chen et al., 2018a).

### Target Gene Prediction of lncRNAs and miRNAs

The function of lncRNAs is mainly realized by their action on target genes in a cis or trans relationship, and cis regulation of lncRNAs and their target mRNAs are based on a location relationship. Trans-regulation was predicted here by calculating the binding energy. In addition, the two correlation coefficients of lncRNA and mRNA were calculated using Spearman and Pearson (Spearman_COR ≥ 0.6 and Pearson_COR ≥ 0.6). A cis relationship was defined as lncRNA within 10 K upstream of mRNA, or within 20 kb downstream of mRNA. If the lncRNA and mRNA binding energy was beyond this range, RNAPlex (Tafer and Hofacker, 2008) was used to analyze the binding energy, and if it was found to be less than −30, it was defined as a trans relationship.

psRobot (Wu et al., 2012), TAPIR (Bonnet et al., 2010), and TargetFinder (Fahlgren and Carrington, 2010) were used to predict the plant targets of the miRNA. Furthermore, GO and KEGG pathway enrichment analyses of the target genes were annotated based on the GO database (Ye et al., 2006) and KEGG database (Kanehisa et al., 2007).

### Identification of ceRNAs Involved in Tre6P Signaling Pathway

A hypergeometric distribution model was used to test whether the DE Tre6P signaling pathway genes shared a significant number of miRNA binding sites with DE lncRNAs. The LF-related ceRNAs were selected according to the following criteria: (1) the DE floral transition pathway coding-genes, lncRNAs, and miRNAs were significantly differentially expressed; (2) the DE floral transition pathway coding-genes shared the same DE miRNA with ceRNA (mRNA/lncRNA) and with the same MRE; and (3) the expression levels of the floral transition pathway coding-genes and predicted ceRNAs were opposite to those of the shared miRNA. The ceRNA network was presented using Cytoscape (version 3.8.0) (Shannon et al., 2003).

### Determining Tre6P Content

Trehalose-6-phosphate (Tre6P) was extracted from the frozen pulverized floral bud tissues of eight randomly selected accessions (four NF and four LF types, 0.1–0.5 g fresh weight), and three biological replicates were performed. Tissue homogenates were extracted for 30 min of ice-cold PBS (pH 7.4) and centrifuged for 15 min at 3,000 × *g* and 4°C. Supernatants were then used for detection of Tre6P content using Plant Trehalose-6-Phosphate (T6P) ELISA Kit (Yan Qi Biological Technology, Shanghai, China). The optical density (OD) of the samples and six standard products was measured at the wavelength of 450 nm with BioTek’s Gen5*™* Microplate Readers (BioTek, Winooski, VT, United States) and calculated based on the standard curve (with a correlation coefficient *R*^2^ ≥ 0.99), which was established using the concentrations and ODs of six standard products (0, 7.5, 15, 30, 60, and 120 pg/ml). The Non-linear Curve Fit was established to build the standard curve using the Origin Pro 2021 software (Supplementary Figure 1). Student’s *t*-test was performed in analysis and conducted using the Origin Pro 2021 software. Data are given as the means ± standard deviation (SD) of three independent biological replicates.

### Quantitative Real-Time PCR Validation of lncRNAs and mRNAs

Total RNA was extracted using GenePure Plus Plantpoly RNA Kit (rich in polysaccharides and polyphenols) (CodonX Biotechnology, Beijing, China). One microgram of DNA-free RNA was transcribed into first-strand cDNA using All-in-One First-Strand Synthesis MasterMix (with dsDNase) (CodonX Biotechnology, Beijing, China). qRT-PCR was conducted with a Roche LightCyler 480 instrument using 2 × SYBR Green qPCR Premix (Universal) (CodonX Biotechnology, Beijing, China). Primers for target genes were designed using Primer 3.0.^1^ The actin gene was used as an internal reference to normalize the qRT-PCR data. All the primers are listed in Supplementary Table 2. Each reaction was performed in triplicate, and the data from real-time PCR amplification were analyzed using the 2^–ΔΔCt^ method. Student’s *t*-test was performed in the analysis and conducted using the Origin Pro 2021 software. The data are presented as the means ± standard deviation (SD) of three independent biological replicates.

## Results

### Morphological Comparisons Between NF and LF Types

**FIGURE 1:**
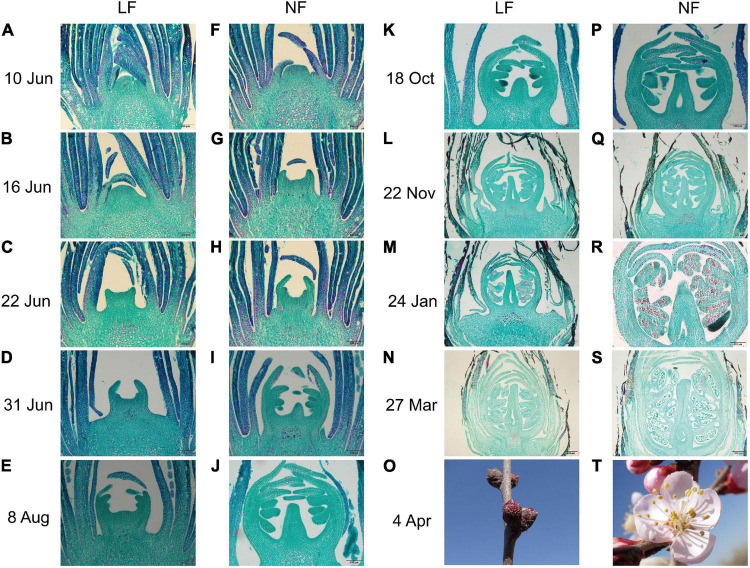
Comparison of the flower bud differentiation process between **(A–E,K–O)** LF and **(F–J,P–T)** NF in *P. sibirica.* **(A)** Undifferentiated stage with buds in the leaf bud state; the growth point is small and tightly wrapped in layers of bud scales. **(B,F)** Initial differentiation stage with the top of the growth point bulges gradually and the bud scales become loose. **(C,G)** Calyx differentiation stage with calyx primordia gradually becoming elongate and bent inward. **(D,H)** Petal differentiation stage with petal primordium as a small second lobe at the bottom of the calyx primordium, wrapped in the calyx primordium and elongate with the calyx primordium. **(E,I)** Stamen differentiation stage with stamen primordium as the third lobe inside, forming a number of protuberances. **(J,K)** Pistil differentiation stage with pistil primordium bulges in the middle of the bottom and continued elongation. **(L,P)** Initiation of ovary and differentiation of sporogenous tissue. **(M,Q)** Initiation of ovule and formation of microspore mother cell. **(N,R)** Differentiation of ovule. **(S)** Formation of pollen grain. **(O)** Flower bud of LF. **(T)** Bloom of NF.

Both NF and LF type accessions were selected to explore the process of floral bud differentiation. The FBD of LF types was found to be delay by 7–15 days compared to that of NF types (Supplementary Table 1). Histological analysis showed that floral bud differentiation was divided into six floral bud differentiation stages, namely, the undifferentiated (Figure 1A), initial differentiation (Figures 1B,F), sepal differentiation (Figures 1C,G), petal differentiation (Figures 1D,H), stamen differentiation (Figures 1E,I), and pistil differentiation (Figures 1J,K) stages, and four reproductive differentiation stages (Figures 1L–N,P–S). Compared with the NF types, the initial differentiation stage was exhibited at least 6 days later in the LF types (Figures 1B,F). All of the floral bud differentiation stages were delayed in the LF types, and this resulted in the LF types remaining in the bud stage while the NF group was blooming (Figures 1O,T).

### Genome-Wide Association Study of Flowering Time

**FIGURE 2:**
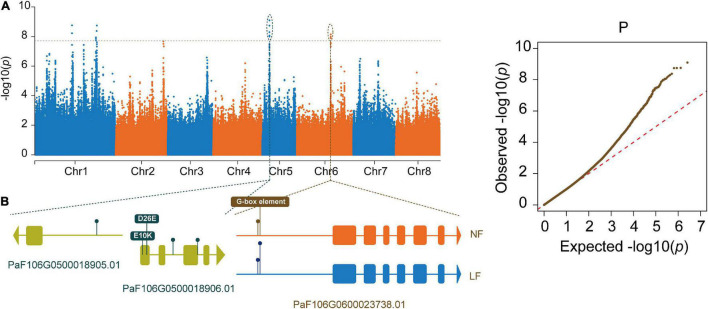
GWAS of flowering time in *Prunus sibirica*. **(A)** Manhattan and quantile–quantile (QQ) plots for GWAS. **(B)** Gene structures of candidate genes. Brown box indicated G-box element in the promoter of *PsTPPF*. Rounded rectangle and triangle represented exons and the last exons, respectively.

To better identify the candidate genes associated with flowering time, we conducted a GWAS for this trait in *P. sibirica*. The phenotypes of 66 accessions were classified into NF (43) and LF (23) types base on the FBD from the 3 years in succession. A total of 2,598,398 high-quality SNPs were identified by mapping against the *P. sibirica* reference genome, and these were used in the subsequent analyses. Principal component analysis (PCA) showed two differentiated clusters, which were consistent with the classifications from the FBD (Supplementary Figure 2). The GWAS on flowering time was conducted using an MLM, and this identified 16 SNPs with 12 predicted genes for the flowering time (Figure 2A), which were located on chromosomes 1, 5, and 6 (Supplementary Table 3). Among the SNPs, seven with three predicted genes (PaF106G0500018905.01, PaF106G0500018906.01, and PaF106G0600023738.01) were located in the gene region (Figure 2 and Supplementary Table 3), including five SNPs on chromosome 5 (Chr. 5: 3872427 bp,3874628 bp, 3874678 bp, 3874988 bp, and 3875280 bp) and two SNPs on chromosome 6 (Chr. 6: 18107193 bp and 18107219 bp). All the seven SNPs were highly associated with the phenotype of flowering time, which could be explained by 86.36–93.94% of the phenotypic variance (Supplementary Table 4). The predicted functions of PaF106G0500018906.01 and PaF106G0600023738.01 were associated with *ubiquitin-conjugating enzyme E2* (*UBC*) and *trehalose-6-phosphate phosphatase* (*TPP*), respectively, whereas the predicted function of PaF106G0500018905.01 was unknown. In particular, an SNP mutation (Chr. 6: 18107219 bp) was found to be located on the promoter of *PsTPPF* (PaF106G0600023738.01), which resulted in a point mutation in the third nucleotide of a G-box core sequence (TACGTG), suggesting that the mutated site might hinder the binding of G-box interaction proteins, thus affecting the transcriptional expression *PsTPPF*. These results will be valuable for the development of molecular markers for late flower breeding in *P. sibirica*.

### Analysis of lncRNA and mRNA in NF and LF Types

To comprehensively understand the regulatory mechanism associated with flowering time, whole-transcriptome RNA sequencing of *P. sibirica* floral buds was conducted, and 515,976,068 raw reads were obtained. After filtering of adaptor sequences and low-quality reads, over 97% clean reads remained (Supplementary Table 5). Approximately 61% of these clean data were mapped to the *P. sibirica* genome (Supplementary Table 5). A total of 28,545 mRNAs, including 24,518 known and 4,027 novel ones, and 3,423 novel lncRNAs were identified (Supplementary Table 6). We found that most of the lncRNAs contained only one exon, and that most of the mRNAs have two exons (Supplementary Figure 3A). The length of the lncRNAs was concentrated at 500 bp, while the mRNAs were 500–1,000 bp in length (Supplementary Figure 3B). We also found that most of the lncRNAs and mRNAs contained only one transcript (Supplementary Figure 3C).

**FIGURE 3:**
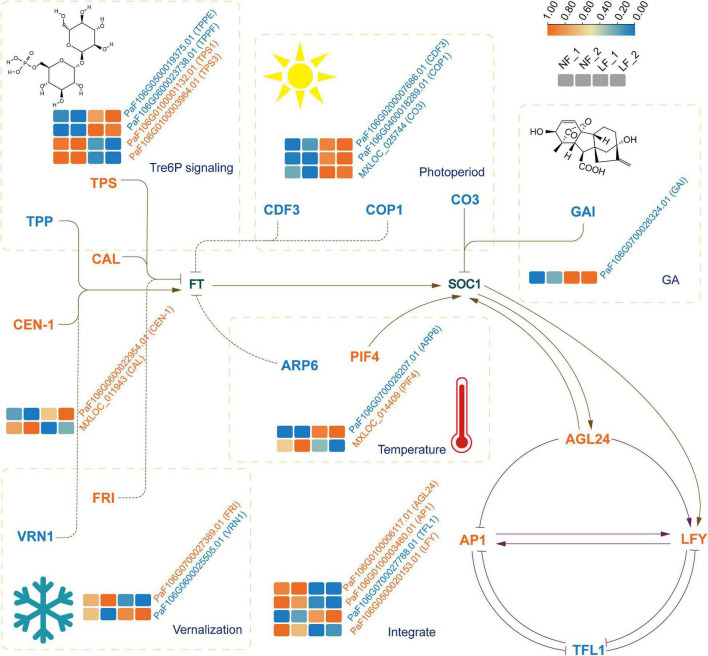
The expression profiles of genes implicated in floral transition from six pathways. The four boxes (left to right) in one row of each heat map correspond to the expression levels in NF_1, NF_2, LF_1, and LF_2, respectively. *TPS, trehalose-6-phosphate synthase, TPP, trehalose-6-phosphate phosphatase, CDF3, cycling dof factor 3; COP1, constitutively photomorphogenic 1; CO3, CONSTANS like 3; GAI, GA insensitive; ARP6, actin related protein 6; PIF4, phytochrome interacting factor 4; FRI, FRIGIDA; VRN1, vernalization 1; AGL24, agamous like24; AP1, apetala1, LFY, LEAFY; TFL1, terminal flower 1.* Full and dashed lines indicated the directly and indirectly acting, respectively. Arrows meant positive relationships and stubs meant the opposite relationships.

We analyzed the expression profiles of the differential expression (DE) mRNAs and lncRNAs. A total of 6,110 DEmRNAs and 1,351 DE lncRNAs were identified. Of these, 3,017 known mRNAs, 586 novel mRNAs, and 731 lncRNAs were upregulated. In contrast, 2,038 known mRNAs, 469 novel mRNAs, and 620 lncRNAs were downregulated (Supplementary Figure 4). Notably, *PsTPPF* was highly expressed in LF than NF types; it was a candidate gene identified by GWAS (Figure 3).

A DE lncRNA-DE mRNA network was constructed according to location relationship and binding sequence. A total of 9,345 pairs of cis-regulatory (Supplementary Table 7) and 1,871 pairs of trans-regulatory genes were identified; of which, 2,723 pairs (1,829 mRNAs and 1,054 lncRNAs) in cis-regulatory relationship and 633 pairs (38 mRNAs and 280 lncRNAs) in trans-regulatory were found to be DE mRNAs–DE lncRNA interactions. GO and KEGG pathway analyses of the putative target DE mRNAs were performed to better understand the potential function of the DE lncRNAs. The GO analysis showed that 852, 1,119, and 871 genes enriched the biological process (BP), cellular component (CC), and molecular function (MF) categories, respectively. Cellular process (GO: 0009987) in the BP category, membrane (GO:0016020) in the CC category, and binding (GO:0005488) in the MFs category were the top subcategories of each category (Supplementary Figure 5A). The most enriched 20 KEGG pathways of the target mRNAs were analyzed, and the most significant enriched pathways were the biosynthesis of secondary metabolites (ko:01110), plant hormone signal transduction (ko: 04075), and starch and sucrose metabolism (ko: 00500) (Supplementary Figure 5B).

### Analysis of sRNA in NF and LF Types

A total of 116,046,350 raw reads were generated from the small RNA libraries (Supplementary Table 8). After filtering for adaptor sequences and low-quality reads, 111,180,013 clean reads remained (Supplementary Table 8), and over 81% of the clean tags were mapped to the reference genome (Supplementary Table 8). We identified 241 conserved miRNAs (Supplementary Figure 6A) and 317 predicted miRNAs (Supplementary Figure 6B). The lengths of the conserved miRNAs ranged from 18 to 24 nucleotides (nt), and 21 nt miRNAs (142) were the most abundant (Supplementary Figure 7A). The lengths of the predicted miRNAs ranged from 19 to 30 nt, and 30 nt miRNAs (95) were the most abundant (Supplementary Figure 7B).

We identified 148 DE miRNAs in LF vs. NF types, including 80 upregulated miRNAs and 68 downregulated miRNAs (Supplementary Figure 8). Among these miRNAs, 352 DE miRNA–DE mRNA interactions, including 37 DE miRNA and 310 DE mRNAs were identified according to the negative regulation between the DE miRNAs and the corresponding DE mRNAs. miR482d-5p_4 was the most abundant miRNA, followed by miR167d, miR395a-3p, and miR396a (Supplementary Figure 9). To understand the potential functions of the DE miRNAs, we conducted GO and KEGG pathway analyses of the putative target DE mRNAs. The GO analysis showed that 187, 404, and 304 genes could be categorized as BP, CC, and MF categories, respectively. The most enriched subcategories of each category were as follows: cellular process (GO:0009987) in the BP category, membrane part (GO:0044425), membrane (GO:0016020) in the CC category, and catalytic activity (GO:0003824) in the MF category (Supplementary Figure 10A). We analyzed the enriched 20 KEGG pathways of the target mRNAs, and the top two significant enriched pathways were found to be metabolic pathways (ko:01100) and the biosynthesis of secondary metabolites (ko:01110) (Supplementary Figure 10B).

### Identification of DE mRNAs and Their Corresponding lncRNAs and miRNAs Involved in Floral Transition Pathways

**FIGURE 4:**
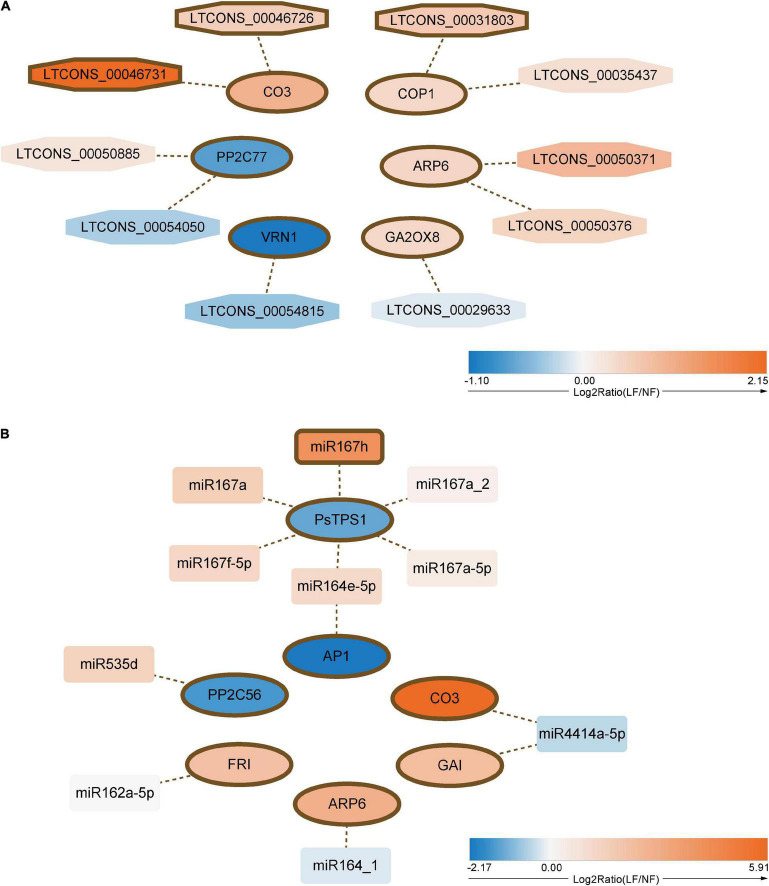
Interaction networks of differential expression (DE) floral transition genes with **(A)** lncRNAs and **(B)** miRNAs. Ellipse, hexagon, and round rectangle indicated the mRNA, lncRNA, and miRNA, respectively. Arbitrary shape with thick border represented differentially expressed.

To understand the relationship between lncRNAs and miRNAs, and flowering time, we further selected 24 DE mRNAs by blasting with known floral transition genes (Figure 3 and Supplementary Table 9), which involved in the photoperiod, vernalization, GA, temperature, and Tre6P signaling pathways. We analyzed the 24 mRNAs and their corresponding miRNAs and lncRNAs. Ten lncRNA–DE mRNA pairs, containing ten lncRNAs and six DE mRNAs, were selected to construct the lncRNA–mRNA network (Figure 4A). Specially and specifically, three DE lncRNA-DE mRNA pairs, COP1 (PaF106G0400018289.01), CCLR (LTCONS_00031803), CO3 (MXLOC_025744), COCLR1 (LTCONS_00046726), CO3 (MXLOC_025744), and COCLR2 (LTCONS_00046731) were positively regulated. In addition, we identified 12 miRNA-mRNA interactions of 24 DE mRNAs (Figure 4B). Notably, miR167h and its target mRNA, *PsTPS1* (PaF106G0100001132.01), encoding trehalose-6-phosphate synthase, were predicted, and these have been previously identified as being involved in the Tre6P signaling pathway of floral transition (Schluepmann et al., 2003).

### Regulation of LF by the Tre6P Signaling Pathway

**FIGURE 5:**
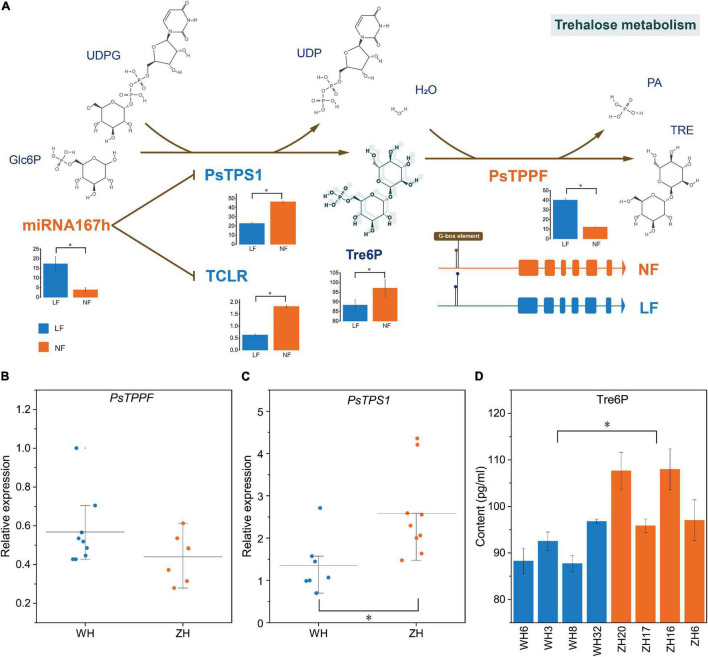
Trehalose-6-phosphate (Tre6P) signaling pathways regulation mechanism. Relative transcript levels of **(A)** *PsTPPF* and **(B)** *PsTPS1*, and content of **(C)** Tre6P in eight randomly selected accessions. **(D)** CeRNA of Tre6P signaling pathway. Trehalose-6-phosphate synthase (TPS) catalyzes glucose-6-phosphate (Glc6P) and UDP-glucose (UDPG) into Tre6P, and then Tre6P is dephosphorylated into trehalose by trehalose-6-phosphate phosphatase (TPP). PsTPS1 was down-regulated by competitively binding miR167h with *TCLR*. The histograms were visualized using FPKM of mRNA and lncRNA and TPM of miRNA. H2O: Water. PA, Phosphoric acid; TRE, Trehalose; *, significant differences *p* < 0.05.

There are three genes associated with the LF types that were obtained from GWAS: PaF106G0500018905.01 and PaF106G0500018906.01 were not expressed in either LF or NF types, but *PsTPPF* was differentially expressed in LF vs. NF types. TPS and TPP can affect floral transition by controlling Tre6P content (Wahl et al., 2013), which is the only product of the enzymatic synthesis reaction of TPS and the only substrate of the enzymatic degradation reaction by TPP. In RNA-seq, *PsTPS1* was expressed at low levels in the LF types, whereas *PsTPPF* was highly expressed in the LF types(Figure 5A). Furthermore, we analyzed the expression profiles of *PsTPS1* and *PsTPPF* in the two types of groups to confirm the result by qRT-PCR, and the expression patterns were found to be consistent with the sequencing results. This suggested that the expression levels of *PsTPS1* and *PsTPPF* may be associated with flowering time in *P. sibirica* (Figures 5B,C). We further found that the Tre6P content in most of the LF-type floral buds was lower than that in the NF types, which indicates that the decrease in Tre6P content in LF was caused by the expression levels of *PsTPS1* and *PsTPPF* (Figure 5D).

### Construction of ceRNA Networks of Tre6P Signaling Pathway

Two candidate DE mRNAs involved in the Tre6P signaling pathway, *PsTPS1* and *PsTPPF*, were identified with an integrative strategy of combining GWAS and whole-transcriptome RNA sequencing analysis. To better understand the gene regulatory network of the LF types, putative DE lncRNA–DE miRNA–DE mRNAs in ceRNA networks were identified. According to the ceRNA hypothesis, the expression levels of lncRNAs and miRNAs, and mRNAs and miRNAs should show negative correlations, and the lncRNAs and mRNAs should show a positive correlation (Tay et al., 2014). We identified a ceRNA network consisting of one mRNA (*PsTPS1*), one miRNA (miR167h, a miR167 family member), and one lncRNA (*TCLR*) (Figure 5A). In the constructed ceRNA network, the expression profiles of *PsTPS1* and *TCLR* were negatively correlated with miR167h. In addition, there was not a ceRNA network of *PsTPPF*.

## Discussion

When flowering in early spring, the yield of *P. sibirica* is reduced when temperatures are low (Dennis, 1979), and promoting the LF trait has become an important breeding objective. In this study, we comprehensive investigated the characterization of floral bud differentiation and found that compared with the NF types, the delay in the initial differentiation stage resulted in delay flowering time in the LF types. Many floral transition genes of *A. thaliana*, *Vitis vinifera*, and *M. domestica* have been found to affect the flowering time of transgenic plants at the time of floral transition (Boss et al., 2006; Tränkner et al., 2010; Wahl et al., 2013).

Floral transition time is one of the factors affecting the flowering time of plants (Blázquez et al., 2001), and it is regulated by a complex network composed of multiple genes that affect flowering time in response to exogenous environmental and endogenous influences (Blázquez et al., 2001). The results of this study show that 24 DE mRNAs of a regulatory network from both environmental and endogenous signals relate to floral transition and likely control the LF trait. Our results show that *COP1* and *CO3* act on photoperiod pathway, and these have been previously reported to affect the flowering time in *A. thaliana* and *Oryza sativa* (Jang et al., 2008; Kim et al., 2008). We also found that *COP1* and *CO3* interacted with three new lncRNAs (*CCLR*, *COCLR1*, and *COCLR2*). The expression patterns of *PIF4* and *ARP6* in regulating LF are in accordance with those of earlier studies conducted on *A. thaliana*, which found *PIF4* and *ARP6* controlling flowering time by activating the expression of *FT* and *FLC*, respectively, in response to warm temperatures (Deal et al., 2005; Kumar et al., 2012). Our results indicate that environmental signals, such as photoperiod and temperature, may be associated with LF in *P. sibirica*. Integration genes, such as *TFL1*, in *Arabis alpina* (Wang et al., 2011), *Populus* (Mohamed et al., 2010), *M. domestica* (Kotoda et al., 2006), Rosa, and *Fragaria vesca* (Iwata et al., 2012) have been found to act as a photoperiod-regulated floral repressor. In this study, *TFL1* was found to be differentially expressed in NF vs. LF types, and this result is supported by those of previous studies. Furthermore, phytohormone metabolic and signaling pathways have previously been proposed as flowering regulators (Ohto et al., 2001; Davis, 2009). For example, *GA2OXs* and *GAI* have been reported to control the flowering time of *Kalanchoë blossfeldiana*, *Petunia hybrida*, and *A. thaliana* (Wilson and Somerville, 1995; Gargul et al., 2013) through GA metabolic and signal pathways, and *FsPP2Cs* negatively regulated the flowering time in *A. thaliana* in the ABA signal pathway (Reyes et al., 2006). These genes were also differentially expressed in NF vs. LF types in our study, and they likely play important roles in controlling the LF trait of *P. sibirica*. In summary, the genes identified here provide information about the regulatory pathways of floral transition, and can be used as genetic resources for developing LF varieties of *P. sibirica*.

Previous studies have shown a correlation between sugar content and floral transition in *A. thaliana* (Ohto et al., 2001; Ortiz-Marchena et al., 2015), *M. domestica* (Li et al., 2018), and *R. chinensis* (Guo et al., 2017). Tre6P is an intermediate product of starch and sucrose metabolism, and it acts both as energy source and signal molecule of flowering time (Schluepmann et al., 2003; Fichtner and Lunn, 2021). Moreover, Tre6P content is regulated by the expression of *TPS* and *TPP* in *A. thaliana* (Schluepmann et al., 2003; Wahl et al., 2013). Two candidate genes (*PsTPPF* and *PsTPS1*) involved in Tre6P signaling pathway were found by GWAS and the whole-transcriptome RNA sequencing analysis in our study, and their expression levels showed inhibitory effects to the content level of Tre6P. However, as floral transition is controlled by an intricate genetic network, it is considered that the other 22 floral transition genes may also regulate the LF trait together with *PsTPPF* and *PsTPS1* in the LF types of *P. sibirica*.

The ceRNA regulatory network has not been widely constructed in plants, and the first ceRNA interaction of *pho2-induced phosphate starvation1* (*IPS1*)-miR399 was reported in *A. thaliana* (Franco-Zorrilla et al., 2007). Therefore, to explore the ceRNA network and functions of the important candidate genes associated with LF in *P. sibirica*, we conducted an integrated analysis of the ceRNAs of *PsTPPF* and *PsTPS1* in the Tre6P signaling pathway. We identified a ceRNA network composed of one interaction ceRNA, one lncRNA (*TCLR*), and one mRNA (*PsTPS1*), which shared with one miRNA (miR167h). We hypothesize that the low expression of *PsTPS1* is negatively regulated by *TCLR* competitively binding to miR167h, and that the high expression of *PsTPPF* is caused by a single SNP located in its promoter, which might result in the LF trait in *P. sibirica*. Studies have shown that miR167a-d played important roles in floral organ development in *A. thaliana* (Wu et al., 2006). However. miR167h may be related with LF through the Tre6P signaling pathway in *P. sibirica*.

We proposed that a new regulatory flowering gene (*PsTPPF*) is related to LF in *P. sibirica*, and we constructed a new ceRNA network (*PsTPS1*, *TCLR*, and miR167h) for the LF types of *P. sibirica*. However, it is also considered that the other 22 differentially expressed floral transition genes with their interactional lncRNAs and miRNAs may also be involved in the regulation of LF of *P. sibirica*, and further studies are, thus, required. This study identified the molecular regulation mechanism associated with LF in *P. sibirica*, and the results can be used as a valuable genetic resource for breeding LF varieties of *P. sibirica*.

## Data Availability Statement

The datasets presented in this study can be found in online repositories. The names of the repository/repositories and accession number(s) can be found in the article/Supplementary Material.

## Author Contributions

TW and LW designed and supervised the experiments. WX conducted the experiments, analyzed the data, and wrote the manuscript. WB, HL, CC, and HB helped analyzed the sequencing data and experiments. WB, MH, GZ, HZ, NG, and YC performed the sample collection. TW, LW, and WX revised the manuscript. All authors read and approved the final version of the manuscript.

## Conflict of Interest

The authors declare that the research was conducted in the absence of any commercial or financial relationships that could be construed as a potential conflict of interest.

## Publisher’s Note

All claims expressed in this article are solely those of the authors and do not necessarily represent those of their affiliated organizations, or those of the publisher, the editors and the reviewers. Any product that may be evaluated in this article, or claim that may be made by its manufacturer, is not guaranteed or endorsed by the publisher.

## Footnotes

1
https://primer3.org/
