# Supplementary material for: Insights Into the Molecular Mechanisms of Late Flowering in Prunus sibirica by Whole-Genome and Transcriptome Analyses
Source: Front Plant Sci. 2022 Jan 25;12:802827. doi: 10.3389/fpls.2021.802827 (PMC8821173; doi:10.3389/fpls.2021.802827)
Supplement: Supplementary file 11 [file Table_1.DOCX]

**Supplementary Table 1.** The full bloom date of 66 *P. sibirica* accessions in 2015 to 2017.

| Sample | Phenotype | Full bloom date (FBD) | | |
| --- | --- | --- | --- | --- |
|  |  | 2015 | 2016 | 2017 |
| WH1 | Late-flowering | 29-Apr | 24-Apr | 15-Apr |
| WH10 | Late-flowering | 25-Apr | 21-Apr | 17-Apr |
| WH101 | Late-flowering | 26-Apr | 22-Apr | 19-Apr |
| WH11 | Late-flowering | 26-Apr | 24-Apr | 16-Apr |
| WH12 | Late-flowering | 25-Apr | 21-Apr | 17-Apr |
| WH127 | Late-flowering | 27-Apr | 24-Apr | 18-Apr |
| WH13 | Late-flowering | 25-Apr | 22-Apr | 19-Apr |
| WH14 | Late-flowering | 25-Apr | 21-Apr | 16-Apr |
| WH15 | Late-flowering | 28-Apr | 23-Apr | 16-Apr |
| WH18 | Late-flowering | 24-Apr | 18-Apr | 16-Apr |
| WH19 | Late-flowering | 26-Apr | 23-Apr | 12-Apr |
| WH20 | Late-flowering | 27-Apr | 23-Apr | 17-Apr |
| WH21 | Late-flowering | 26-Apr | 24-Apr | 17-Apr |
| WH22 | Late-flowering | 25-Apr | 21-Apr | 16-Apr |
| WH23 | Late-flowering | 26-Apr | 20-Apr | 16-Apr |
| WH24 | Late-flowering | 27-Apr | 20-Apr | 15-Apr |
| WH25 | Late-flowering | 24-Apr | 20-Apr | 17-Apr |
| WH28 | Late-flowering | 23-Apr | 18-Apr | 15-Apr |
| WH29 | Late-flowering | 25-Apr | 20-Apr | 17-Apr |
| WH3 | Late-flowering | 25-Apr | 18-Apr | 16-Apr |
| WH30 | Late-flowering | 24-Apr | 18-Apr | 17-Apr |
| WH31 | Late-flowering | 26-Apr | 22-Apr | 18-Apr |
| WH32 | Late-flowering | 25-Apr | 20-Apr | 18-Apr |
| WH33 | Late-flowering | 25-Apr | 20-Apr | 18-Apr |
| WH34 | Late-flowering | 27-Apr | 22-Apr | 18-Apr |
| WH37 | Late-flowering | 24-Apr | 23-Apr | 19-Apr |
| WH39 | Late-flowering | 27-Apr | 23-Apr | 19-Apr |
| WH4 | Late-flowering | 28-Apr | 25-Apr | 16-Apr |
| WH40 | Late-flowering | 28-Apr | 24-Apr | 14-Apr |
| WH41 | Late-flowering | 26-Apr | 21-Apr | 14-Apr |
| WH42 | Late-flowering | 24-Apr | 20-Apr | 19-Apr |
| WH43 | Late-flowering | 27-Apr | 21-Apr | 13-Apr |
| WH45 | Late-flowering | 26-Apr | 21-Apr | 18-Apr |
| WH48 | Late-flowering | 25-Apr | 23-Apr | 18-Apr |
| WH5 | Late-flowering | 23-Apr | 23-Apr | 19-Apr |
| WH51 | Late-flowering | 24-Apr | 22-Apr | 19-Apr |
| WH6 | Late-flowering | 23-Apr | 23-Apr | 18-Apr |
| WH7 | Late-flowering | 26-Apr | 22-Apr | 19-Apr |
| WH8 | Late-flowering | 25-Apr | 22-Apr | 19-Apr |
| WH86 | Late-flowering | 26-Apr | 22-Apr | 18-Apr |
| WH88 | Late-flowering | 27-Apr | 23-Apr | 18-Apr |
| WH9 | Late-flowering | 25-Apr | 20-Apr | 19-Apr |
| WH92 | Late-flowering | 27-Apr | 23-Apr | 18-Apr |
| ZH1 | Normal-flowering | 18-Apr | 12-Apr | 4-Apr |
| ZH10 | Normal-flowering | 18-Apr | 12-Apr | 4-Apr |
| ZH11 | Normal-flowering | 18-Apr | 12-Apr | 4-Apr |
| ZH12 | Normal-flowering | 18-Apr | 12-Apr | 4-Apr |
| ZH13 | Normal-flowering | 18-Apr | 12-Apr | 4-Apr |
| ZH14 | Normal-flowering | 18-Apr | 12-Apr | 4-Apr |
| ZH15 | Normal-flowering | 18-Apr | 12-Apr | 4-Apr |
| ZH16 | Normal-flowering | 18-Apr | 12-Apr | 4-Apr |
| ZH17 | Normal-flowering | 18-Apr | 12-Apr | 4-Apr |
| ZH18 | Normal-flowering | 18-Apr | 12-Apr | 4-Apr |
| ZH19 | Normal-flowering | 18-Apr | 12-Apr | 4-Apr |
| ZH2 | Normal-flowering | 18-Apr | 12-Apr | 4-Apr |
| ZH20 | Normal-flowering | 18-Apr | 12-Apr | 4-Apr |
| ZH21 | Normal-flowering | 18-Apr | 12-Apr | 4-Apr |
| ZH22 | Normal-flowering | 18-Apr | 12-Apr | 4-Apr |
| ZH23 | Normal-flowering | 18-Apr | 12-Apr | 4-Apr |
| ZH3 | Normal-flowering | 18-Apr | 12-Apr | 4-Apr |
| ZH4 | Normal-flowering | 18-Apr | 12-Apr | 4-Apr |
| ZH5 | Normal-flowering | 18-Apr | 12-Apr | 4-Apr |
| ZH6 | Normal-flowering | 18-Apr | 12-Apr | 4-Apr |
| ZH7 | Normal-flowering | 18-Apr | 12-Apr | 4-Apr |
| ZH8 | Normal-flowering | 18-Apr | 12-Apr | 4-Apr |
| ZH9 | Normal-flowering | 18-Apr | 12-Apr | 4-Apr |
