# Supplementary material for: Insights Into the Molecular Mechanisms of Late Flowering in Prunus sibirica by Whole-Genome and Transcriptome Analyses
Source: Front Plant Sci. 2022 Jan 25;12:802827. doi: 10.3389/fpls.2021.802827 (PMC8821173; doi:10.3389/fpls.2021.802827)
Supplement: Supplementary file 12 [file Table_2.DOCX]

**Supplementary Table 2.** The primers were used for qRT-PCR in this study.

| Gene Name | Gene ID | Forward Primer Sequence (5'-3') | Reverse Primer Sequence (5'-3') | Amplicon Size (bp) |
| --- | --- | --- | --- | --- |
| Actin* | PaF106G0604497700.01 | GTGCCTGCCATGTATGTTGCCA | CAGTGGTGGTGAACATGTACCC | 226 |
| *PsTPS1* | PaF106G0100001132.01 | ATTCAGAGTTGTTGCTTTGGGAC | TTTGTCCACCGAAGCCTGAG | 139 |
| *PsTPPF* | PaF106G0600023738.01 | CTGCCATAGTGAGTGGAAGATG | GAACCTTTGGCTGGACCTTTA | 112 |

*: the primers were described by Balogh et, al. Doi: 10.3389/fpls.2019.00402
