# Supplementary material for: Insights Into the Molecular Mechanisms of Late Flowering in Prunus sibirica by Whole-Genome and Transcriptome Analyses
Source: Front Plant Sci. 2022 Jan 25;12:802827. doi: 10.3389/fpls.2021.802827 (PMC8821173; doi:10.3389/fpls.2021.802827)
Supplement: Supplementary file 13 [file Table_3.DOCX]

**Supplementary Table 3.** The candidate genes of flowing time from GWAS.

| Chromosome | Peak | Ref | Alt | Value | Gene Start | Gene End | Gene ID | Location site | Annotation |
| --- | --- | --- | --- | --- | --- | --- | --- | --- | --- |
| Chr1 | 32611900 | A | G | 7.853 | 32609344 | 32611879 | PaF106G0100005061.01 | Intergenic | subtilisin-like protease SBT1.9 |
| Chr1 | 32612543 | T | C | 7.943 |  |  |  |  |  |
| Chr1 | 32973080 | C | T | 8.377 | 32973623 | 32975714 | PaF106G0100005135.01 | Intergenic | patatin-like protein 2 |
| Chr1 | 33077549 | T | G | 8.744 | 33076660 | 33077521 | PaF106G0100005152.01 | Intergenic | transcription factor MYB44-like |
| Chr1 | 19864578 | C | T | 8.225 | 19859804 | 19859231 | PaF106G0100002665.01 | Intergenic | myb family transcription factor PHL8-like |
|  |  |  |  |  | 19873218 | 19874667 | PaF106G0100002666.01 | Intergenic | null |
| Chr1 | 19869333 | C | T | 8.755 | 19859804 | 19859231 | PaF106G0100002665.01 | Intergenic | myb family transcription factor PHL8-like |
|  |  |  |  |  | 19873218 | 19874667 | PaF106G0100002666.01 | Intergenic | null |
| Chr1 | 32615277 | C | G | 7.743 | 32609344 | 32611879 | PaF106G0100005061.01 | Intergenic | subtilisin-like protease SBT1.9 |
|  |  |  |  |  | 32617745 | 32619007 | PaF106G0100005062.01 | Intergenic | heat stress transcription factor B-4 |
| Chr5 | 3872427 | C | A | 8.297 | 3871394 | 3871864 | PaF106G0500018905.01 | promoter | null |
| Chr5 | 3874628 | G | A | 8.739 | 3874574 | 3875602 | PaF106G0500018906.01 | Non-synonymous coding | ubiquitin-conjugating enzyme E2 32-like |
| Chr5 | 3874678 | C | G | 8.004 |  |  |  | Non-synonymous coding |  |
| Chr5 | 3874988 | C | A | 9.098 |  |  |  | intronic |  |
| Chr5 | 3875280 | C | A | 8.053 |  |  |  | intronic |  |
| Chr5 | 3889994 | C | T | 8.067 | 3879912 | 3880012 | PaF106G0500018909.01 | Intergenic | uncharacterized LOC18777620 |
|  |  |  |  |  | 3897897 | 3898029 | PaF106G0500018910.01 | Intergenic | ubiquitin-conjugating enzyme E2 32-like |
| Chr6 | 18104140 | T | A | 8.148 | 18082651 | 18084566 | PaF106G0600023737.01 | Intergenic | ethylene-responsive transcription factor RAP2-4 |
|  |  |  |  |  | 18108294 | 18111032 | PaF106G0600023738.01 | Intergenic | trehalose-6-phosphate phosphatase |
| Chr6 | 18107193 | C | A | 7.990 | 18082651 | 18084566 | PaF106G0600023737.01 | Intergenic | ethylene-responsive transcription factor RAP2-4 |
|  |  |  |  |  | 18108294 | 18111032 | PaF106G0600023738.01 | promoter | trehalose-6-phosphate phosphatase |
| Chr6 | 18107219 | C | T | 7.990 | 18082651 | 18084566 | PaF106G0600023737.01 | Intergenic | ethylene-responsive transcription factor RAP2-4 |
|  |  |  |  |  | 18108294 | 18111032 | PaF106G0600023738.01 | promoter | trehalose-6-phosphate phosphatase |

Ref: Reference alleles, Alt: Alternate non-reference alleles
