# Supplementary material for: Insights Into the Molecular Mechanisms of Late Flowering in Prunus sibirica by Whole-Genome and Transcriptome Analyses
Source: Front Plant Sci. 2022 Jan 25;12:802827. doi: 10.3389/fpls.2021.802827 (PMC8821173; doi:10.3389/fpls.2021.802827)
Supplement: Supplementary file 14 [file Table_4.DOCX]

**Supplementary Table 4.** Genotypes associated with the LF trait in *P. sibirica* accessions.

| Sample | Phenotype | Location | | | | | | |
| --- | --- | --- | --- | --- | --- | --- | --- | --- |
|  | Normal: 0,  Late: 1 | Chr. 5: 3872427 | Chr. 5: 3874628 | Chr. 5: 3874678 | Chr. 5: 3874988 | Chr. 5: 3875280 | Chr. 6: 18107193 | Chr. 6: 18107219 |
| REF |  | C | G | C | C | C | C | C |
| WH1 | 0 | AA | AA | GG | AA | AA | AA | TT |
| WH10 | 0 | CA | GA | CG | CA | NN | CA | CT |
| WH101 | 0 | AA | AA | GG | AA | AA | AA | TT |
| WH11 | 0 | AA | AA | GG | AA | AA | AA | TT |
| WH12 | 0 | AA | AA | GG | AA | AA | AA | TT |
| WH127 | 0 | AA | AA | GG | AA | AA | CA | TT |
| WH13 | 0 | AA | AA | GG | AA | AA | CA | CT |
| WH14 | 0 | CA | GG | CC | CC | CA | AA | TT |
| WH15 | 0 | AA | AA | GG | AA | AA | CA | CT |
| WH18 | 0 | AA | AA | GG | AA | AA | AA | TT |
| WH19 | 0 | AA | AA | GG | AA | AA | CC | CC |
| WH20 | 0 | CA | GA | CG | CA | CA | AA | TT |
| WH21 | 0 | CA | GA | CG | CA | CA | AA | TT |
| WH22 | 0 | CA | GA | CG | CA | AA | CA | CT |
| WH23 | 0 | AA | AA | GG | AA | AA | CC | CC |
| WH24 | 0 | CA | GA | CG | CA | CA | CA | CT |
| WH25 | 0 | AA | AA | GG | AA | AA | CC | CC |
| WH28 | 0 | AA | AA | GG | AA | AA | CA | CT |
| WH29 | 0 | AA | AA | GG | AA | AA | AA | TT |
| WH3 | 0 | CA | GG | CC | CC | CC | CA | CT |
| WH30 | 0 | CA | GA | CG | CA | AA | CA | CT |
| WH31 | 0 | AA | AA | CG | AA | AA | AA | TT |
| WH32 | 0 | AA | AA | GG | AA | AA | AA | TT |
| WH33 | 0 | AA | AA | GG | AA | AA | CA | CT |
| WH34 | 0 | AA | AA | GG | AA | AA | CA | CT |
| WH37 | 0 | CA | GA | CC | CA | AA | CA | CT |
| WH39 | 0 | AA | AA | GG | AA | AA | AA | TT |
| WH4 | 0 | AA | AA | GG | AA | AA | AA | TT |
| WH40 | 0 | CC | GG | CC | CC | AA | AA | TT |
| WH41 | 0 | CA | GA | CG | CA | AA | AA | TT |
| WH42 | 0 | AA | AA | GG | AA | NN | AA | TT |
| WH43 | 0 | CA | GG | CC | CC | CC | CA | CT |
| WH45 | 0 | AA | AA | GG | AA | AA | AA | TT |
| WH48 | 0 | AA | AA | GG | AA | AA | AA | TT |
| WH5 | 0 | AA | AA | GG | AA | AA | AA | TT |
| WH51 | 0 | CA | GA | CG | CA | CA | CC | CC |
| WH6 | 0 | CA | GA | CG | AA | AA | AA | TT |
| WH7 | 0 | AA | AA | GG | AA | AA | AA | TT |
| WH8 | 0 | AA | AA | GG | AA | AA | AA | TT |
| WH86 | 0 | AA | AA | GG | AA | AA | CA | CT |
| WH88 | 0 | AA | AA | GG | AA | AA | CA | CT |
| WH9 | 0 | CA | GA | CG | CA | AA | CA | CT |
| WH92 | 0 | AA | GA | CG | CA | CA | CA | CT |
| ZH1 | 1 | CC | GG | CC | CC | CC | CC | CC |
| ZH10 | 1 | CC | GG | CC | CC | CC | CC | CC |
| ZH11 | 1 | AA | GG | CC | CC | CC | CC | CC |
| ZH12 | 1 | CC | GG | CC | CC | CC | CC | CC |
| ZH13 | 1 | CC | GG | CC | CC | AA | CC | CC |
| ZH14 | 1 | CC | GG | CC | CC | CC | CC | CC |
| ZH15 | 1 | CC | GG | CC | CC | CC | CC | CC |
| ZH16 | 1 | CA | GG | CC | CC | CC | CC | CC |
| ZH17 | 1 | CC | GG | CC | CC | CC | CC | CC |
| ZH18 | 1 | CC | GG | CC | CC | CA | CC | CC |
| ZH19 | 1 | CC | GA | CG | CA | CA | CC | CC |
| ZH2 | 1 | CC | GG | CC | CC | CC | CC | CC |
| ZH20 | 1 | CC | GG | CC | CC | CC | CC | CC |
| ZH21 | 1 | CC | GG | CC | CC | CC | CC | CC |
| ZH22 | 1 | CC | GG | CC | CC | CC | CC | CC |
| ZH23 | 1 | CC | GG | CC | CC | CC | CC | CC |
| ZH3 | 1 | CA | GG | CC | CC | CC | CC | CC |
| ZH4 | 1 | CC | GA | CC | CA | CA | CC | CC |
| ZH5 | 1 | CC | GG | CC | CC | CC | CC | CC |
| ZH6 | 1 | CC | GG | CC | CC | CA | CC | CC |
| ZH7 | 1 | CC | GG | CC | CC | AA | CC | CC |
| ZH8 | 1 | CC | GG | CC | CC | CC | CC | CC |
| ZH9 | 1 | CC | GG | CC | CC | CC | CC | CC |
