# Supplementary material for: Insights Into the Molecular Mechanisms of Late Flowering in Prunus sibirica by Whole-Genome and Transcriptome Analyses
Source: Front Plant Sci. 2022 Jan 25;12:802827. doi: 10.3389/fpls.2021.802827 (PMC8821173; doi:10.3389/fpls.2021.802827)
Supplement: Supplementary file 15 [file Table_5.DOCX]

**Supplementary Table 5.** Statistical summary of the four libraries.

| Sample | Total Raw Reads | Total Clean Reads | Total Clean Base | Total Mapping Ratio | Uniquely Mapping Ratio | Q20 of Fq1 | Q20 of Fq2 | Q30 of Fq1 | Q30 of Fq2 |
| --- | --- | --- | --- | --- | --- | --- | --- | --- | --- |
| WH_1 | 128994044 | 126024156 | 18903623400 | 61.82% | 59.93% | 98.77% | 95.05% | 96.41% | 89.47% |
| WH_2 | 128993990 | 125935092 | 18890263800 | 62.80% | 60.94% | 98.74% | 95.19% | 96.38% | 89.79% |
| ZH_1 | 128994232 | 126692018 | 19003802700 | 60.32% | 58.45% | 98.69% | 95.03% | 96.24% | 89.43% |
| ZH_2 | 128993802 | 126398370 | 18959755500 | 61.21% | 59.33% | 98.75% | 95.22% | 96.38% | 89.80% |
| Total | 515976068 | 505049636 | 75757445400 |  |  |  |  |  |  |

Q20: The percentage of bases with a Phred value > 20

Q30: The percentage of bases with a Phred value > 30

Fq1: read1

Fq2: read2

Clean Reads Ratio: Total Clean Reads / Total Raw Reads
