# Supplementary material for: Insights Into the Molecular Mechanisms of Late Flowering in Prunus sibirica by Whole-Genome and Transcriptome Analyses
Source: Front Plant Sci. 2022 Jan 25;12:802827. doi: 10.3389/fpls.2021.802827 (PMC8821173; doi:10.3389/fpls.2021.802827)
Supplement: Supplementary file 16 [file Table_6.DOCX]

**Supplementary Table 6.** Statistics for the number of expressed RNAs.

| Sample | mRNAs | Know mRNAs | Novel mRNAs | lncRNAs | Novel lncRNAs |
| --- | --- | --- | --- | --- | --- |
| WH_1 | 26010 | 22135 | 3875 | 2987 | 2987 |
| WH_2 | 26028 | 22155 | 3873 | 3002 | 3002 |
| ZH_1 | 25903 | 22036 | 3867 | 2944 | 2944 |
| ZH_1 | 25828 | 21974 | 3854 | 2978 | 2978 |
| Total | 28545 | 24518 | 4027 | 3423 | 3423 |
