# Supplementary material for: Insights Into the Molecular Mechanisms of Late Flowering in Prunus sibirica by Whole-Genome and Transcriptome Analyses
Source: Front Plant Sci. 2022 Jan 25;12:802827. doi: 10.3389/fpls.2021.802827 (PMC8821173; doi:10.3389/fpls.2021.802827)
Supplement: Supplementary file 17 [file Table_7.DOCX]

**Supplementary Table 7.** Target relationships between lncRNAs and mRNAs.

| Start | Region | Target Pair Number | mRNA Number | lncRNA Number | Flag |
| --- | --- | --- | --- | --- | --- |
| overlap | | 1463 | 1220 | 1037 | Upstream |
| 1 | 1-1000 | 378 | 346 | 344 | Upstream |
| 1001 | 1001-2000 | 320 | 294 | 289 | Upstream |
| 2001 | 2001-3000 | 269 | 251 | 249 | Upstream |
| 3001 | 3001-4000 | 253 | 236 | 238 | Upstream |
| 4001 | 4001-5000 | 263 | 267 | 242 | Upstream |
| 5001 | 5001-6000 | 316 | 283 | 287 | Upstream |
| 6001 | 6001-7000 | 266 | 240 | 242 | Upstream |
| 7001 | 7001-8000 | 262 | 235 | 238 | Upstream |
| 8001 | 8001-9000 | 237 | 217 | 215 | Upstream |
| 9001 | 9001-10000 | 251 | 236 | 229 | Upstream |
| 1 | 1-1000 | 337 | 367 | 337 | Downstream |
| 1001 | 1001-2000 | 282 | 275 | 282 | Downstream |
| 2001 | 2001-3000 | 282 | 274 | 282 | Downstream |
| 3001 | 3001-4000 | 273 | 279 | 273 | Downstream |
| 4001 | 4001-5000 | 277 | 272 | 277 | Downstream |
| 5001 | 5001-6000 | 248 | 259 | 248 | Downstream |
| 6001 | 6001-7000 | 261 | 264 | 261 | Downstream |
| 7001 | 7001-8000 | 293 | 304 | 293 | Downstream |
| 8001 | 8001-9000 | 262 | 258 | 262 | Downstream |
| 9001 | 9001-10000 | 258 | 254 | 258 | Downstream |
| 10001 | 10001-11000 | 250 | 251 | 250 | Downstream |
| 11001 | 11001-12000 | 244 | 252 | 244 | Downstream |
| 12001 | 12001-13000 | 256 | 258 | 256 | Downstream |
| 13001 | 13001-14000 | 230 | 228 | 230 | Downstream |
| 14001 | 14001-15000 | 261 | 256 | 261 | Downstream |
| 15001 | 15001-16000 | 271 | 266 | 271 | Downstream |
| 16001 | 16001-17000 | 264 | 280 | 264 | Downstream |
| 17001 | 17001-18000 | 251 | 254 | 251 | Downstream |
| 18001 | 18001-19000 | 257 | 263 | 257 | Downstream |
| 19001 | 19001-20000 | 254 | 248 | 254 | Downstream |
