# Supplementary material for: Insights Into the Molecular Mechanisms of Late Flowering in Prunus sibirica by Whole-Genome and Transcriptome Analyses
Source: Front Plant Sci. 2022 Jan 25;12:802827. doi: 10.3389/fpls.2021.802827 (PMC8821173; doi:10.3389/fpls.2021.802827)
Supplement: Supplementary file 18 [file Table_8.DOCX]

**Supplementary Table 8.** Statistical summary of the four small RNA libraries.

| Sample | Raw tag count | Clean tag count | Percentage (%) | Mapped tag | Percentage (%) |
| --- | --- | --- | --- | --- | --- |
| WH_1 | 28699389 | 27571744 | 96.07 | 22652787 | 82.16 |
| WH_2 | 29331934 | 28045347 | 95.61 | 23020120 | 82.08 |
| ZH_1 | 29973158 | 28639803 | 95.55 | 23420328 | 81.78 |
| ZH_2 | 28041869 | 26923119 | 96.01 | 21882183 | 81.28 |
| Total | 116046350 | 111180013 |  | 90975418 |  |
