# Supplementary material for: Insights Into the Molecular Mechanisms of Late Flowering in Prunus sibirica by Whole-Genome and Transcriptome Analyses
Source: Front Plant Sci. 2022 Jan 25;12:802827. doi: 10.3389/fpls.2021.802827 (PMC8821173; doi:10.3389/fpls.2021.802827)
Supplement: Supplementary file 19 [file Table_9.DOCX]

**Supplementary Table 9.** Differential expression (DE) mRNAs of floral transition.

| Pathways | Name | Gene ID | log2Ratio (LF/NF) | Up/Down Regulation (LF/NF) | p-value | adjust p-value | comment |
| --- | --- | --- | --- | --- | --- | --- | --- |
| Photoperiod | *CO3* | MXLOC_025744 | 2.748935844 | Up | 7.37E-21 | 6.24E-21 | Negative regulation *FT-like* (Kim et al., 2008) |
|  | *COP1* | PaF106G0400018289.01 | 1.247513598 | Up | 3.36E-44 | 4.61E-44 | E3 ubiquitin ligase to ubiquitinate *CO* (Jang et al., 2008) |
|  | *CDF3* | PaF106G0200007686.01 | 1.998008945 | Up | 7.44E-23 | 6.67E-23 | Repress transcription of *CO* (Corrales et al., 2017;Xu et al., 2021) |
| Vernalization | *FRI* | PaF106G0600025505.01 | 1.01716608 | Up | 9.05E-07 | 4.45E-07 | Up-regulate *FLC* (Clarke and Dean, 1994) |
|  | *VRN1* | PaF106G0700027389.01 | -2.172147542 | Down | 4.19E-11 | 2.55E-11 | Maintain the methylated state of *FLC* chromatin (Levy et al., 2002) |
| GA | *GAI* | PaF106G0700026324.01 | 1.065515765 | Up | 2.44E-161 | 1.00E-160 | Mutation delay flowering (Wilson and Somerville, 1995) |
|  | *GA3OX* | PaF106G0200007638.01 | -2.878557113 | Down | 2.37E-07 | 1.20E-07 | DELLA protein, repress flowering (Mitchum et al., 2006) |
|  | *GA2OX* | PaF106G0400017368.01 | 1.158629846 | Up | 1.35E-173 | 5.98E-173 | Reduce the level of active GA, over-expression delay flowering (Gargul et al., 2013) |
|  | *GA2OX2* | PaF106G0100001314.01 | 1.445877837 | Up | 0.0000114 | 0.00000521 |  |
|  | *GA2OX8* | MXLOC_016189 | 1.22928841 | Up | 2.33E-167 | 9.93E-167 |  |
| Tre6p | *PsTPS1* | PaF106G0100001132.01 | -1.033840041 | Down | 0 | 0 | Catalyze Tre6P, mutation sustain vegetative growth (Wahl et al., 2013) |
|  | *PsTPS3* | PaF106G0100003964.01 | -1.035529893 | Down | 0 | 0 |  |
|  | *PsTPPE* | PaF106G0500019375.01 | 1.409139347 | Up | 1.37E-16 | 1.02E-16 | Degrade Tre6P, over-expression delay flowering (Wahl et al., 2013) |
|  | *PsTPPF* | PaF106G0600023738.01 | 1.725424819 | Up | 0 | 0 |  |
| Integrate | *AGL24* | PaF106G0100006117.01 | -9.551728755 | Down | 1.43E-66 | 2.69E-66 | Induce *SOC1* (Michaels et al., 2003) |
|  | *LFY* | PaF106G0500020153.01 | -1.386040861 | Down | 1.39E-12 | 8.98E-13 | Integrate signal from GA and photoperiod pathway (Blázquez et al., 1997) |
|  | *TFL1* | PaF106G0700027788.01 | 2.048216907 | Up | 7.13E-24 | 6.56E-24 | Repress *AP1* and *LFY* with each other (Hanano and Goto, 2011) |
| Temperature | *PIF4* | MXLOC_014409 | -2.663850673 | Down | 4.49E-24 | 4.15E-24 | Control the thermosensory activation of flowering, induce *FT* (Kumar et al., 2012;Thines et al., 2014) |
|  | *ARP6* | PaF106G0700026207.01 | 1.337111061 | Up | 1.24E-46 | 1.77E-46 | Maintain *FLC* expression (March-Díaz et al., 2007) |
|  | *PP2C56* | PaF106G0700026499.01 | -1.221479217 | Down | 0 | 0 | Sensitive of ABA, inhibition of *SnRK* (Tsai and Gazzarrini, 2012;Rodrigues et al., 2013) |
|  | *PP2C77* | PaF106G0700026694.01 | -1.438016992 | Down | 0 | 0 |  |
|  | *AP1* | PaF106G0100003460.01 | -1.697452567 | Down | 2.66E-69 | 5.18E-69 | Active by *LFY* (Bowman et al., 1993;Kotoda et al., 2000) |
|  | *CAL* | MXLOC_011943 | -1.05316395 | Down | 1.31E-09 | 7.49E-10 | Negative regulation *FT* (Cheng et al., 2021) |
|  | *CEN-1* | PaF106G0600022954.01 | 2.291367888 | Up | 4.70E-07 | 2.34E-07 | Over-expression delay transition to flowering (Amaya et al., 1999) |

Amaya, I., Ratcliffe, O.J., and Bradley, D.J. (1999). Expression of CENTRORADIALIS (CEN) and CEN-like genes in tobacco reveals a conserved mechanism controlling phase change in diverse species. *The Plant Cell* 11**,** 1405-1417.

Blázquez, M.A., Soowal, L.N., Lee, I., and Weigel, D. (1997). LEAFY expression and flower initiation in Arabidopsis. *Development* 124**,** 3835-3844.

Bowman, J.L., Alvarez, J., Weigel, D., Meyerowitz, E.M., and Smyth, D.R. (1993). Control of flower development in Arabidopsis thaliana by APETALA1 and interacting genes. *Development* 119**,** 721-743.

Cheng, S., Chen, P., Su, Z., Ma, L., Hao, P., Zhang, J., Ma, Q., Liu, G., Liu, J., and Wang, H. (2021). High‐resolution temporal dynamic transcriptome landscape reveals a GhCAL‐mediated flowering regulatory pathway in cotton (Gossypium hirsutum L.). *Plant biotechnology journal* 19**,** 153-166.

Clarke, J.H., and Dean, C. (1994). Mapping FRI, a locus controlling flowering time and vernalization response in Arabidopsis thaliana. *Molecular and General Genetics MGG* 242**,** 81-89.

Corrales, A.R., Carrillo, L., Lasierra, P., Nebauer, S.G., Dominguez‐Figueroa, J., Renau‐Morata, B., Pollmann, S., Granell, A., Molina, R.V., and Vicente‐Carbajosa, J. (2017). Multifaceted role of cycling DOF factor 3 (CDF3) in the regulation of flowering time and abiotic stress responses in Arabidopsis. *Plant, cell & environment* 40**,** 748-764.

Gargul, J., Mibus, H., and Serek, M. (2013). Constitutive overexpression of Nicotiana GA 2 ox leads to compact phenotypes and delayed flowering in Kalanchoë blossfeldiana and Petunia hybrida. *Plant Cell, Tissue and Organ Culture (PCTOC)* 115**,** 407-418.

Hanano, S., and Goto, K. (2011). Arabidopsis TERMINAL FLOWER1 is involved in the regulation of flowering time and inflorescence development through transcriptional repression. *The Plant Cell* 23**,** 3172-3184.

Jang, S., Marchal, V., Panigrahi, K.C., Wenkel, S., Soppe, W., Deng, X.W., Valverde, F., and Coupland, G. (2008). Arabidopsis COP1 shapes the temporal pattern of CO accumulation conferring a photoperiodic flowering response. *The EMBO journal* 27**,** 1277-1288.

Kim, S.-K., Yun, C.-H., Lee, J.H., Jang, Y.H., Park, H.-Y., and Kim, J.-K. (2008). OsCO3, a CONSTANS-LIKE gene, controls flowering by negatively regulating the expression of FT-like genes under SD conditions in rice. *Planta* 228**,** 355-365.

Kotoda, N., Wada, M., Komori, S., Kidou, S.-I., Abe, K., Masuda, T., and Soejima, J. (2000). Expression pattern of homologues of floral meristem identity genes LFY and AP1 during flower development in apple. *Journal of the American Society for Horticultural Science* 125**,** 398-403.

Kumar, S.V., Lucyshyn, D., Jaeger, K.E., Alós, E., Alvey, E., Harberd, N.P., and Wigge, P.A. (2012). Transcription factor PIF4 controls the thermosensory activation of flowering. *Nature* 484**,** 242-245.

Levy, Y.Y., Mesnage, S., Mylne, J.S., Gendall, A.R., and Dean, C. (2002). Multiple roles of Arabidopsis VRN1 in vernalization and flowering time control. *Science* 297**,** 243-246.

March-Díaz, R., García-Domínguez, M., Florencio, F.J., and Reyes, J.C. (2007). SEF, a new protein required for flowering repression in Arabidopsis, interacts with PIE1 and ARP6. *Plant physiology* 143**,** 893-901.

Michaels, S.D., Ditta, G., Gustafson‐Brown, C., Pelaz, S., Yanofsky, M., and Amasino, R.M. (2003). AGL24 acts as a promoter of flowering in Arabidopsis and is positively regulated by vernalization. *The Plant Journal* 33**,** 867-874.

Mitchum, M.G., Yamaguchi, S., Hanada, A., Kuwahara, A., Yoshioka, Y., Kato, T., Tabata, S., Kamiya, Y., and Sun, T.P. (2006). Distinct and overlapping roles of two gibberellin 3‐oxidases in Arabidopsis development. *The Plant Journal* 45**,** 804-818.

Rodrigues, A., Adamo, M., Crozet, P., Margalha, L., Confraria, A., Martinho, C., Elias, A., Rabissi, A., Lumbreras, V., and González-Guzmán, M. (2013). ABI1 and PP2CA phosphatases are negative regulators of Snf1-related protein kinase1 signaling in Arabidopsis. *The Plant Cell* 25**,** 3871-3884.

Thines, B.C., Youn, Y., Duarte, M.I., and Harmon, F.G. (2014). The time of day effects of warm temperature on flowering time involve PIF4 and PIF5. *Journal of Experimental Botany* 65**,** 1141-1151.

Tsai, A.Y.L., and Gazzarrini, S. (2012). AKIN10 and FUSCA3 interact to control lateral organ development and phase transitions in Arabidopsis. *The Plant Journal* 69**,** 809-821.

Wahl, V., Ponnu, J., Schlereth, A., Arrivault, S., Langenecker, T., Franke, A., Feil, R., Lunn, J.E., Stitt, M., and Schmid, M. (2013). Regulation of flowering by trehalose-6-phosphate signaling in Arabidopsis thaliana. *Science* 339**,** 704-707.

Wilson, R.N., and Somerville, C.R. (1995). Phenotypic suppression of the gibberellin-insensitive mutant (gai) of Arabidopsis. *Plant Physiology* 108**,** 495-502.

Xu, D., Li, X., Wu, X., Meng, L., Zou, Z., Bao, E., Bian, Z., and Cao, K. (2021). Tomato SlCDF3 Delays Flowering Time by Regulating Different FT-Like Genes Under Long-Day and Short-Day Conditions. *Frontiers in plant science* 12**,** 826.
